# Supplementary material for: High Prevalence of Astigmatism in Children after School Suspension during the COVID-19 Pandemic Is Associated with Axial Elongation
Source: Children (Basel). 2022 Jun 19;9(6):919. doi: 10.3390/children9060919 (PMC9245603; doi:10.3390/children9060919)
Supplement: Supplementary file 1 [file children-09-00919-s001.zip › children-1749230-supplementary.pdf]

Risk factors of myopia in Hong Kong primary school students

Start of Block: Personal Information about Your Child

The ocular parameters and questionnaire information will be kept strictly confidential and will be used only for research purposes. If you agree to participate in our study (vision screening and questionnaire), please choose "Yes".

○ Yes

○ No

Page Break

Personal Information about Your Child

*

Q1 **Your child's name is (Family name/ First name, e.g. Chan Tai Man):**

____

Q2 **Please choose your child's gender:**

○ Male

○ Female

*

Q3 **Please provide your child's date of birth (YYYY/MM/**DD**, e.g. 2000/01/31):**

____

Q5 **Please choose your child's grade level:**
Click to input choice

▼ 1 ... 6

Page Break

End of Block: Personal Information about Your Child

Start of Block: Information about Time outside School Hours

Q1
During the school lockdown period,
Does your child spend time on the following activities on a weekday (including time for tutorial classes)?

	Yes	No	Don't know
Printed materials (reading, writing, drawing, etc)	○	○	○
Television	○	○	○
Computer (desktop/ laptop)	○	○	○
Tablet computer	○	○	○
Smartphone	○	○	○

Page Break

*

Q2 <u>On the weekday **during the school lockdown period,**</u>
how long does your child spend totally on following activities (including time for tutorial classes)?

	Hours/day (e.g. 0.5)
Printed materials (reading, writing, drawing, etc)	
Television	
Computer (desktop/ laptop)	
Tablet computer	
Smartphone	

Page Break

Q3
During the school lockdown period,
Does your child spend time on following activities (including time for tutorial classes) on the weekend?

	Yes	No	Don't know
Printed materials (reading, writing, drawing, etc)	○	○	○
Television	○	○	○
Computer (desktop/ laptop)	○	○	○
Tablet computer	○	○	○
Smartphone	○	○	○

Page Break

*

Q4

<u>On the weekend</u> **<u>during the school lockdown period,</u>**,

Does your child spend time on following activities (including time for tutorial classes)?

	Hours/day (e.g. 0.5)
Printed materials (reading, writing, drawing, etc)	
Television	
Computer (desktop/ laptop)	
Tablet computer	
Smartphone	

Page Break

End of Block: Information about Time outside School Hours

Start of Block: Information about Outdoor Time outside School Hours

Q84 **Information about Outdoors outside School Hours**

Outdoor physical activities include playing sports, running, riding a bike, etc.
Outdoor leisure activities include walking, having a picnic, etc.

*

Q1 During the school lockdown period (from Monday to Friday),
how long does your child spend on following activities?

	Hours in total (e.g. 2)
Outdoor physical activities	
Outdoor leisure activities	

*

Q2 During the school lockdown period (Saturday and Sunday), how long does your child spend on following activities?

	Hours in total (e.g. 2)
Outdoor physical activities	
Outdoor leisure activities	

Page Break

End of Block: Information about Outdoor Time outside School Hours

Start of Block: Personal Information about Parents

Personal Information about Biological Parents

Q1 **Please choose father's ethnicity:**

- ○ Chinese
- ○ Non-Chinese, please specify (e.g. Filipino, Indonesian, Caucasian): ____
- ○ Mixed, please specify ____

Q2 **Please choose mother's ethnicity:**

- ○ Chinese
- ○ Non-Chinese, please specify (e.g. Filipino, Indonesian, Caucasian): ____
- ○ Mixed, please specify ____

Page Break

Q3 **The highest education attainment of father:**

- ○ Primary or below
- ○ Lower Secondary
- ○ Upper Secondary
- ○ Post-secondary or above

Q4 **The highest education attainment of mother:**

- ○ Primary or below
- ○ Lower Secondary
- ○ Upper Secondary
- ○ Post-secondary or above

Page Break

Q5 **Total monthly family income is (including all sources):**

- ≤HK$ 9,999
- HK$ 10,000 to HK$ 14,999
- HK$ 15,000 to HK$ 19,999
- HK$ 20,000 to HK$ 24,999
- HK$ 25,000 to HK$ 29,999
- HK$ 30,000 to HK$ 39,999
- HK$ 40,000 to HK$ 59,999
- HK$ 60,000 to HK$ 79,999
- ≥HK$ 80,000
- N/A

Page Break

Q6 Has the child's father ever had his vision tested?

- Yes
- No
- Don't know

Page Break

Q7 **Does the child's father suffer from myopia (near-/short-sightedness)?**

◯ Yes

◯ No

◯ Don't know

Page Break

Q8 **Does the child's father have 6.00 D or more myopia (if he had myopia surgery before, is original myopia degree 6.00 D or above) ?**

◯ Yes

◯ No

◯ Don't know

Page Break

Q9 Has the child's mother ever had her vision tested?

◯ Yes

◯ No

◯ Don't know

Page Break

Q10 **Does the child's mother suffer from myopia (near-/short-sightedness)?**

- ○ Yes
- ○ No
- ○ Don't know

Page Break

Q11 **Does the child's mother have 6.00 D or more myopia (**if she had myopia surgery before, is original myopia degree 6.00 D or above**)?**

- ○ Yes
- ○ No
- ○ Don't know

Page Break

End of Block: Personal Information about Parents
